# Absence of change in the gray matter volume of patients with ulcerative colitis in remission: a voxel based morphometry study

**DOI:** 10.1186/s13030-014-0028-7

**Published:** 2015-01-07

**Authors:** Alessandro Agostini, Massimo Campieri, Angela Bertani, Antonella Scarcelli, Daniela Ballotta, Carlo Calabrese, Fernando Rizzello, Paolo Gionchetti, Paolo Nichelli, Francesca Benuzzi

**Affiliations:** Department of Psychology, University of Bologna, Bologna, Italy; Department of Clinical Medicine, IBD Unit, S. Orsola-Malpighi Hospital, University of Bologna, Bologna, Italy; Department of Biomedical, Metabolic Sciences, and Neurosciences, Nuovo Ospedale Civile S. Agostino-Estense, University of Modena and Reggio Emilia, Modena, Italy; Department of Gastroenterology, IBD Unit, Policlinico Hospital, Modena, Italy

**Keywords:** Ulcerative Colitis, Voxel based morphometry, Gray matter volume

## Abstract

**Background:**

Recent neuroimaging studies have investigated the brain involvement in patients with Crohn's disease (CD) and Ulcerative Colitis (UC). Functional studies found abnormalities in cognitive and emotional functions in CD and UC, while a voxel based morphometry (VBM) study found morphological changes in CD. We conducted a VBM study to compare the gray matter (GM) volume of UC patients and controls.

**Methods:**

Eighteen UC patients in remission and eighteen healthy controls underwent structural MRI. VBM is a fully automated technique allowing identification of regional differences in the amount of GM, which enables an objective analysis of the whole brain. VBM was used for comparisons between patients and controls.

**Results:**

UC patients were all in remission and had a mild clinical course. There were no differences between patients and controls in GM volume.

**Conclusion:**

The brain morphology of patients with UC in remission is similar to controls. The lack of GM abnormalities in UC patients might reflect the mild clinical course of the inflammatory bowel disorder. Further research involving patients with different degrees of disease severity or during flares could shed more light on potential brain structural changes in UC.

## Background

Ulcerative colitis (UC) and Crohn’s disease (CD) are chronic, relapsing and remitting disorders of the bowel collectively labelled as inflammatory bowel diseases (IBD). The main symptoms of IBD include bloody diarrhoea, abdominal pain, fever, and weight loss. IBD are associated with stress [1,2], emotional disturbances [3-5], psychological disorders, and impaired cognitive functioning [6]. The plausible neural substrate for these comorbidities have been investigated in recent research focused on the brain involvement in IBD patients [2,5,7]. In patients with CD, both functional and morphological neuroimaging studies have been conducted. Indeed, in these patients, a recent case-control, functional magnetic resonance imaging (fMRI) study has shown impaired neural activity in the mid temporal lobe, in the insula, putamen, and cerebellum in response to stressful stimuli [2]. In addition, brain morphological abnormalities were found in CD patients in a voxel based morphometry (VBM) study [7]. VBM is a fully automated technique enabling an objective study of the whole brain that allows identification of differences in the amount of grey matter (GM) [8]. Although the underlining mechanisms for the brain GM changes in CD need to be thoroughly elucidated, pain and inflammatory mediators have been implicated [7]. We hypothesized that these factors might promote GM changes in UC patients.

In patients with UC, despite an fMRI study that has shown dysfunction in the emotional processing characterized by abnormalities in the activity of the amygdala, thalamic and cerebellar regions [5], brain morphological studies still have not been conducted.

We performed an explorative MRI study using VBM to compare the GM volume of eighteen UC patients in remission and eighteen healthy controls.

## Methods

### Participant recruitment

This study was approved by the local Ethics Committee and all participants signed an informed consent statement. Physicians attending the IBD Unit of the Policlinico-Hospital in Modena examined and enrolled all participants. Eligible UC patients were consecutively recruited when they met the inclusion and exclusion criteria. Clinical and endoscopic data were obtained for all enrolled patients from routinely conducted visits and colonoscopies. All participants were screened for neurological and psychiatric disorders using a semi-structured interview based on the diagnostic and statistical manual of mental disorders (DSM IV).

#### Inclusion criteria

Age > 18, < 65; UC diagnosed at least one year prior to enrollment; clinical remission for at least 6 months; right-handed.

Patients with active disease or recent flares were excluded because medications used during flares, such as steroids [9] and opioids [10], may induce acute GM changes.

#### Exclusion criteria

Signs of disease activity (based on clinical and endoscopic data); use of corticosteroids, biologics, and psychotropic medications in the previous 6 months; current or prior history of neurological, psychiatric disease; chronic pain syndromes; claustrophobia; presence of metallic implants in the body.

At recruitment we collected: age, gender, education, disease location, extra-intestinal manifestations, smoking history, history of intestinal surgery (including appendectomy), clinical colitis activity index (CAI) [11], endoscopic parameters of disease activity, treatment with biologics, maintenance treatments, IBS symptoms.

Patients were asked to rate the severity of pain symptoms at recruitment and during flares. Two visual analogue scales (VAS) were used in which 0 corresponded to "no pain", 3 to "mild pain", 6 to "moderate pain", and 10 to "worst possible pain".

##### Control group

A group of 18 healthy subjects (10 female) were recruited with advertisements from among the staff of Bologna and Modena universities. They underwent the same screening procedures as the UC patients.

#### Neuroimaging protocol

Scanning was performed at the Department of Biomedical, Metabolic and Neurological sciences of Modena University, Baggiovara Hospital. T1- weighted images were acquired using a 3 Tesla Philips Intera system. A SPGR pulse sequence [echo time (TE) = 4.6, repetition time (TR) = 9.9 ms] was used. One hundred seventy contiguous slices were acquired (voxel size = 1 × 1 × 1 mm). VBM was used to identify GM changes between groups. The Matlab 7.1 and SPM8 (Wellcome Trust Centre for Neuroimaging, London, UK) software were used for data analysis.

##### VBM procedure

*Preprocessing:* The pre-processing procedure was conducted according to the vbm8 toolbox (http://dbm.neuro.uni-jena.de/vbm/vbm8-for-spm8/) as implemented in SPM8. The optimized method for vbm8 comprised several consecutive steps: Normalization, segmentation, modulation, and smoothing. The normalization process transforms all the images to the same stereotactic space. This is achieved by registering each of the images to the same template image, the Montreal Neurological Institute/International Consortium for Brain Mapping (MNI/ICBM) 152 standard using linear transformations. The images were segmented in GM, white matter (WM), and cerebrospinal fluid (CSF) volumes. With respect to this pre-processing step, vbm8 differs from the standard VBM procedure. Indeed, segmentation is performed without tissue priors, thus increasing classification accuracy. Volumes were then modulated with Jacobian determinants. Modulation involves scaling by the amount of contraction, so that the total amount of GM in the modulated GM volume remains the same, as it would be in the original images. Finally, the GM images were smoothed by convolving with an isotropic Gaussian kernel (12 × 12 × 12 mm).

*Whole brain analysis*: The total GM volumes of normalized-modulated-smoothed images were compared between groups using a two sample *T*-test. To remove the confounding effects of different brain sizes, we used modulated images that corrected for non-linear warping only. In addition, we modelled the total amount of GM, WM, and CSF as nuisance parameters (additive effects). Modulation is recommended if interested in volume changes rather than differences in concentration (or density). The statistical threshold was set at p < 0.05 family wise error (FWE) corrected.

*Power analysis*: A power analysis was conducted using the software frmipower testing at an alpha-threshold of *p* < 0.05 [12].

### Statistical analysis for non-imaging variables

Statistics were carried out using SPSS software (Chicago, IL). *T*-test was used for comparisons between the two groups and *χ*^2^ (chi-square) for categorical variables.

## Results

### Participants

None of the participants were smokers at recruitment. No socio-demographic differences were observed between patients (N = 18) and controls (N = 18) (Table 1).

**Table 1:** **Socio-demographic and clinical characteristics of the two study groups**

	***UC patients*** ***N = 18***	***Controls*** ***N = 18***	***P***
***Socio-demographics***			
Age (years)	39.06 (±9.64)	36.89 (±11.01)	0.54
Sex (male/female)	10/8	10/8	1
Education (years)	12.56 (±2.17)	14.28 (±4.39)	0.15
***Clinical characteristics***			
Duration of illness (years)	10.33 (6.59)		
Duration of remission (years)	3.94 (4.65)		
Age at diagnosis	28.89 (9.73)		
Location	Proctitis/Distal Colitis 11		
	Left-sided Colitis 7		
	Extensive Colitis 0		
Extraintestinal manifestations	None 18		
Previous surgery	Previously treated 0		
	Never treated 18		
Use of biologics	Previously treated 0		
	Never treated 18		

Values denote mean (±Standard Deviation) or numbers of subjects.

### Patient characteristics

Endoscopic and clinical data were available for all patients. Patients were all in clinical remission and rated the pain "mild" (1.66 ± 0.48, range 1-2) at recruitment. From the diagnosis, 15 patients had 1 flare and 3 had no flares. During flares pain was rated “severe” (7.94 ± 0.80, range 7-10). All patients were in maintenance treatment with 5-amino-salicylic-acid agents. No patients had IBS symptoms. The clinical characteristics of the patients are shown in Table 1.

### VBM

There were no differences in the GM volume of the patients and controls when using the total intracranial volume (TIV) and age as covariates of non-interest.

A power analysis was therefore performed using the previously detected regions of significantly decreased GM volume of CD patients and controls (7) as regions of interest (ROIs). The calculation revealed that the probability of detecting a significant effect in each ROI was the following: Right and left superior and medial frontal gyrus (MNI coordinates: 4 45 35), left middle and superior frontal gyrus (MNI coordinates: -22 25 5), right middle and superior frontal gyrus (MNI coordinates: 27 53 12) power: 0.65; right anterior cingulate (MNI coordinates: 4 28 20) power: 0.58.

## Discussion

In this study, the GM volume of UC patients in remission and healthy controls was compared using VBM. The brain involvement of IBD patients has been investigated in both functional and structural neuroimaging studies [2,5,7]. FMRI studies conducted for patients with UC and CD found abnormalities in emotional and cognitive functions [2,5], while brain morphological changes were found in CD patients in a recent VBM study [7].

In the present study, compared to controls, brain morphological changes did not emerge in UC patients. In patients with CD, the brain volumetric changes were characterized by GM volume reductions in the midcingulate cortex and in a portion of the frontal lobe. In addition, the GM volumes of the cortical and subcortical areas were negatively correlated with disease duration, which suggests progressive volumetric changes in brain areas involved in the nociception, emotional, and cognitive functions of patients [7]. Overall, in VBM studies, a decrease in GM could be due to neural or glial cell apoptosis, decreased cell size, decreased dendritic density, or changes in interstitial fluid. In CD, the chronicity of the disorder, pain, and inflammatory mediators have been implicated in the GM volumetric changes [7,13]. Indeed, the chronic abdominal pain associated to the inflammatory activity determines abundant inputs towards the pain matrix, the distributed network of brain structures involved in nociception. This overstimulation, in turn, might induce neural loss, a phenomenon known as excito-toxicity [13].

Furthermore, in IBD, the overproduction of inflammatory cytokines is believed to promote GM changes by inducing apoptosis in astrocytes and oligodendrocytes, decreased neurogenesis and increased glutamatergic activation, and oxidative stress [14,15]. Although circulating cytokines cannot cross the blood brain barrier, they can propagate inflammatory signals within the brain through the activation of the endothelial and glial cells or perivascular macrophages [16]. Finally, intestinal inflammatory signals may reach the brain via the brain gut axis, the anatomo-functional substrate for bidirectional communications between the brain and the gastrointestinal tract [17,18]. Indeed, the afferent fibers of the vagus nerve are also a target of cytokines [19,20].

The patients with UC enrolled for this study were all in remission and overall had a mild clinical course. Indeed, they had no more than one inflammatory exacerbation from the diagnosis, and none underwent surgery, had extraintestinal manifestations, or were treated with biologics. In addition, pain was mild at recruitment and pain symptoms were judged severe by all patients only during flares. Therefore, the lack of GM abnormalities in UC patients might reflect the mild clinical course of the inflammatory bowel disorder rather than differences in the brain involvement between UC and CD. The enrollment of patients without severe UC or not complaining of persistent pain symptoms limited the investigation of the possible impact of these clinical parameters in the development of potential brain structural changes in UC. Furthermore, the results of the power analysis suggest that the number of enrolled UC patients represents another limitation of the present study.

## Conclusion

In conclusion, the GM changes previously found in a group of CD patients [7] were not replicated in the present explorative VBM study, conducted with patients with UC in remission and of mild clinical course. These findings suggest a key role for the severity of the bowel disorder in the development of the morphological neural changes of IBD patients.

Further research involving an increased number of UC patients is needed to shed more light on the brain involvement in UC. Moreover, the enrollment of patients with a severe disease course or active disease could pave the way to further studies aimed at investigating the plausible neural substrate for the development of emotional disturbances or impaired cognitive functions in IBD.

### Ethics committee

Ethics Committee University Hospital of Modena. Title: Study of brain function with functional magnetic resonance imaging. Practice number 100/01. Supervisor: Professor Paolo Nichelli.

## Abbreviations

CSF: Cerebrospinal fluid
CAI: Clinical colitis activity index
CD: Crohn’s disease
DSM: Diagnostic and statistical manual of mental disorders
TE: Echo time
FWE: Family wise error
fMRI: Functional magnetic resonance imaging
GM: Grey matter
IBD: Inflammatory bowel diseases
MNI/ICBM: Montreal Neurological Institute/International Consortium for Brain Mapping
TR: Repetition time
TIV: Total intracranial volume
UC: Ulcerative colitis
VAS: Visual analogue scales
VBM: Voxel based morphometry
WM: White matter
